# Metabolic Targeting of Breast Cancer Cells With the 2-Deoxy-D-Glucose and the Mitochondrial Bioenergetics Inhibitor MDIVI-1

**DOI:** 10.3389/fcell.2018.00113

**Published:** 2018-09-11

**Authors:** Federico Lucantoni, Heiko Dussmann, Jochen H. M. Prehn

**Affiliations:** ^1^Department of Physiology and Medical Physics, Royal College of Surgeons in Ireland, Dublin, Ireland; ^2^Centre for Systems Medicine, Royal College of Surgeons in Ireland, Dublin, Ireland

**Keywords:** Warburg effect, breast Cancer, MDIVI-1, cell death, bioenergetics, OXPHOS

## Abstract

Breast cancer cells have different requirements on metabolic pathways in order to sustain their growth. Triple negative breast cancer (TNBC), an aggressive breast cancer subtype relies mainly on glycolysis, while estrogen receptor positive (ER+) breast cancer cells possess higher mitochondrial oxidative phosphorylation (OXPHOS) levels. However, breast cancer cells generally employ both pathways to sustain their metabolic needs and to compete with the surrounding environment. In this study, we demonstrate that the mitochondrial fission inhibitor MDIVI-1 alters mitochondrial bioenergetics, at concentrations that do not affect mitochondrial morphology. We show that this effect is accompanied by an increase in glycolysis consumption. Dual targeting of glycolysis with 2-deoxy-D-glucose (2DG) and mitochondrial bioenergetics with MDIVI-1 reduced cellular bioenergetics, increased cell death and decreased clonogenic activity of MCF7 and HDQ-P1 breast cancer cells. In conclusion, we have explored a novel and effective combinatorial regimen for the treatment of breast cancer.

## Introduction

Metabolic rewiring in breast cancer cells critically contributes to disease progression (Long et al., 2016). In general, this malignancy displays a highly lipogenic phenotype with glucose and glutamine playing a central role in sustaining cell growth (Mishra and Ambs, 2015). Breast cancer cells possess a highly versatile metabolism and can use different energy sources and metabolic pathways for their energetic and anabolic requirements. Metabolic rewiring also allows breast cancer cells to adapt to different nutrient available in the surrounding environment and to switch metabolic pathways to adapt to limiting growth conditions (Vander Heiden and DeBerardinis, 2017). It is widely accepted that glucose plays an important role in cancer progression. In 1920, the German scientist Otto Warburg, observed that tumors take up increased amount of glucose, compared to the surrounding cells, with the subsequent fermentation to lactate (Warburg et al., 1927). Of note, breast cancer is a heterogeneous disease that can be divided into several molecular subtypes, each of them characterized by the presence of distinct metabolic alterations. In line with the Warburg effect, it has been shown that triple negative breast cancer (TNBC) and HER2 positive breast cancer possess higher levels of glycolytic activity than estrogen receptor positive (ER+) breast cancer cells (Choi et al., 2013; Pelicano et al., 2014; Lanning et al., 2017). In TNBC, it was reported that EGF signaling is responsible for the activation of the first step in glycolysis (Lim et al., 2016), and that c-Myc regulate this metabolic feature by suppressing the expression of thioredoxin-interacting protein (Shen et al., 2015). Glutamine is also an important mediator of breast cancer metabolism. Indeed, both TNBC and HER2+ subtypes possess increased glutamine consumption and glutaminolysis levels (Kung et al., 2011; Kim et al., 2013; Lampa et al., 2017). In contrast, ER+ breast cancer cells have been found to be more dependent on OXPHOS, even when glycolysis is functioning (Rodriguez-Enriquez et al., 2010; Lanning et al., 2017).

In a recent study, we have recently shown that breast cancer bioenergetics are also controlled by members of the BCL-2 protein family, a family of proteins originally shown to be primarily involved in apoptosis regulation (Czabotar et al., 2014). However, it is now becoming increasingly evident, that BCL-2 family proteins also regulate mitochondrial fusion and fission dynamics and may regulate mitochondrial respiratory chain activity (Chen and Pervaiz, 2007; Alavian et al., 2011; Hardwick and Soane, 2013; Gross, 2016; Williams et al., 2016). BCL2 and BCL(X)L selective inhibitors (Venetoclax and WEHI-539, respectively) were able to decrease mitochondrial bioenergetics and ATP production (Lucantoni et al., 2018). Of note, this metabolic inhibition observed was independent of apoptosis induction. Additionally, we have also shown that these inhibitors were able to decrease mitochondrial morphology and fusion/fission dynamics (Lucantoni et al., 2018). We then highlighted that dual targeting of glycolysis, with 2-deoxy-D-glucose, and mitochondrial metabolism, using BCL2 inhibitors can be used as a potential strategy to stop the progression of both ER+ and TN breast cancer (Lucantoni et al., 2018).

In the present study, we wanted to explore the effect of mitochondrial dynamics interference on bioenergetics and cell survival. Mitochondria are tightly regulated organelles that provides for different cellular functions, apart from their role of producing high amount of energy (Friedman and Nunnari, 2014). The complex mitochondrial structure is deeply linked to the bioenergetics function performed by this organelle at the physiological level (Galloway et al., 2012). The highly mitochondrial plasticity is regulated by enzymatic processes (Westermann, 2010). Mitochondrial fusion and fission are important in maintaining a healthy pool of mitochondria, since structural abnormalities lead to bioenergetics defects (Chen et al., 2005), compromised autophagy control systems (Twig and Shirihai, 2011) and accumulation of mitochondrial DNA damage (Chen et al., 2010). These membrane remodeling activities are mediated by dynamin-related proteins 1 (Drp1), mitofusin (Mfn) 1 and 2 and Opa1. Drp1 is the key regulator of mitochondrial fission, the splitting of one mitochondrion in two or more smaller mitochondria. When recruited on the outer mitochondrial membrane by human fission protein 1 (Fis1), Drp1, together with other structural bending proteins, forms oligomeric rings around the membrane to fragment it (Detmer and Chan, 2007; McBride and Soubannier, 2010). Due to the importance of mitochondrial dynamics, an effort has been made to develop chemical tools to alter these processes. In fact, a selective Drp1 inhibitor, MDIVI-1, has been discovered through a screening of a chemical library (Cassidy-Stone et al., 2008). Nonetheless, a recent work, shed light on MDIVI-1 selectivity and mechanism of action, as this compound was reported to inhibit reversibly mitochondrial complex I-dependent O_2_ consumption (Bordt et al., 2017).

Here, we show, by employing population and single cell time-lapse imaging approaches, how the dual targeting of mitochondrial bioenergetics, with the fission and complex I inhibitor MDIVI-1, and glycolysis inhibition, can be used as a potential strategy for the treatment of breast cancer.

## Materials and Methods

### Materials and Reagents

Fetal bovine serum, RPMI 1640 medium, Thiazolyl Blue Tetrazolium Bromide (MTT), dimethyl sulfoxide (DMSO), sodium pyruvate, D-glucose, 2-deoxy-D-glucose and MDIVI-1 came from Sigma-Aldrich (Dublin, Ireland). Tetramethylrhodamine methyl ester (TMRM) was from Invitrogen (Biosciences, Ireland).

### Cell Lines

MCF7 and HDQ-P1 cells were cultured in RPMI-1640 supplemented with 10% FBS, 1% L-Glutamine and 1% Penicilin/Streptomycin. All cell lines were incubated at 37°C in humidified atmosphere with 5% of CO_2_. Cell lines were authenticated by STR typing from Source Bioscience (Nottingham, United Kindom).

### MTT Assay

The MTT assay was used to determine mitochondrial activity following combination treatment of MDIVI-1 and 2DG. MCF7 cells and HDQ-P1 cells were seeded at a density of 1.5 × 10^4^ cells for well on 96-well plates, kept at 5% CO_2_ and 37°C and treated with increasing concentration of MDIVI-1 (from 0.1 to 10 μM) in combination with 2DG (from 0.3 to 30 mM). After 72 h, 20 μL of 5 mg/mL MTT in 1X PBS was added to each well and the plate incubated at 37°C for 4 h. Consequently medium was removed and crystals were suspended in 100 μL DMSO. Absorbance at 570 nm was recorded on a Clariostar reader (BMG Labtech, Ireland). Experiments were repeated three times on cultures from different platings; each treatment was performed in triplicate during every experiment.

### Live Cell Time-Lapse Imaging of Mitochondrial and Cytosolic ATeam FRET Probe, TMRM Dye, and Glucose FRET Probe

Cells were seeded at a concentration of 2 × 10^3^ in sterile Willco dishes and let to adhere over-night. Then, the plasmid with the mitochondrial targeted Ateam construct (Imamura et al., 2009) was transfected into MCF7 cells with lipofectamine 2000 for 4 h. On the day of the experiment, adherent cells were washed twice with krebs-hepes buffer (KB, 140 mM NaCl, 5.9 mM KCl, 1.2 mM MgCl_2_, 15 mM HEPES) and the medium replaced with 1 mL of KB containing 30 nM TMRM, 2 mM sodium pyruvate and 2.5 mM CaCl_2_. Mineral oil was added on top of the KB to prevent evaporation and the dishes transferred to a heated stage above a 63×/1.4 NA Plan-Apochromat oil immersion objective lens on an inverted confocal laser-scanning microscopes (LSM 710, Zeiss). Mitochondrial ATP kinetics measurements were carried out using a lasers of 405, 488, and 561 nm for excitation of FRET/CFP, YFP and TMRM respectively with a pixel dwell time of 2.55 μs and images taken every minute. Detection ranges were set to 445–513 nm and 513–562 nm for CFP and FRET/YFP, while 562–710 nm was used for TMRM with pinholes set to 2 μm optical sectioning (FWHM). Cells were treated with 0.1, 1, and 10 μM MDIVI-1, with a time window of 20 min before each addition.

The FLII^12^Pglu-700μδ6 “glucose-FRET” probe (plasmid #17866, Addgene) was used to detect intracellular glucose concentration with a linear response range between 0.05 and 9.6 mM. Glucose binds to the glucose–galactose-binding protein with the subsequent probe conformational change and increase in FRET signal. This plasmid has been optimized to reduce pH sensitivity [enhanced yellow fluorescent protein (YFP) replaced with citrine protein] and other potential artifacts (Fehr et al., 2003; Takanaga et al., 2008). Again, cells were seeded in sterile Willco dishes and transfected as described above. On the day of the experiment KB with 5 mM glucose was added on top of the cells, with mineral oil to prevent evaporation. The dishes were transferred to a heated stage above a 40×/1.3 Numerical Aperture (NA) Plan-Neofluar oil immersion objective lenses on a inverted epifluorescence microscope (Axiovert 200M, Zeiss), used with selected polychroic mirror and filter wheel settings. Experiments were carried out using 0.09% of a HBO 100 mercury short-arc lamp for excitation with a band pass of 438/24 nm (center wavelength and band width) for FRET/CFP (cyan fluorescent protein) and a band pass filter with 500/24 nm YFP (yellow fluorescent protein) with exposure time of 20 ms, and 531/40 nm for TMRM with an exposure time of 10 ms. Baseline levels were measured for 20 min, then 1 μM MDIVI-1 was added for 30 min. Subsequently we added 5 mM 2DG for 30 min and 20 mM glucose for the last 20 min. Same setup and settings were used for cytosolic ATP reporter imaging using ATeam1.03-nD/nA/pcDNA3 (plasmid #51958, Addgene). For this experiment, we measured 20 min baseline followed by 5 mM 2DG treatment for 30 min. Then we added 3 μM MDIVI-1 or vehicle for 2 h, and 20 mM glucose for the final 30 min.

Images were processed using ImageJ2 (National Institutes of Health, Bethesda, MD, United States) and Metamorph 7.5 (Universal Imaging Co., Westchester, PA, United States). Time-lapse sequences were imported into ImageJ and background was first subtracted from each image. After creating combined images of the three fields of views for each channel sequence, a median filter with a radius of one pixel was applied. The combined images were then processed using Metamorph. Mitochondria, cytosolic glucose and ATP signals within cells were segmented from background using the YFP time lapse images. The segmented mitochondrial or cytosolic areas were converted into a mask used to remove background values from any further analysis of the FRET/CFP stack. To this end the FRET image stack was first multiplied by the YFP-mask and divided by CFP image stack, and regions of interest were then selected for analysis. A custom made Metamorph journal was used to obtain the average intensity signal from all regions, and an excel macro was then applied to sort the values and to converted them to percentage normalized to the baseline. All experiments were performed at least three times independently of each other.

### Live Cell Time-Lapse Imaging of Caspase DEVD FRET Probe

MCF7-DEVD cells (Rehm et al., 2002) where plated on glass bottom dishes (Willco Wells, Netherlands). Time lapse experiments where performed on LSM 710 or a home build epifluorescence live cell imaging setup both equipped with stage incubator set to 37°C and 5% CO_2_. Drugs were added as described in the figures (10 mM 2DG after 30 min and 3 μM MDIVI-1 after 60 min) and cells were imaged in intervals of 2–5 min for 48 h. On the LSM 710 and the epifluorescence settings were used as described above in order to image the CFP and YFP intensities to determine a disruption of FRET monitoring caspase-3 activation. Single cell CFP/FRET kinetics were analyzed after background subtraction in all YFP positive areas using ImageJ (1.51 k, by Wayne Rasband, NIH) and plotted in MS-Excel. An increase in the CFP/FRET ratio is indicative of DEVD substrate cleavage. Treatment and control experiments were performed three times each.

#### Synergy Calculations

MTT was employed to measure mitochondrial activity, while phenol red absorbance was used to obtain pH values of the nutrient medium covering the live cells. After 72 h treatment MTT protocol was utilized as previously described. An excel template was used to calculate the mitochondrial activity after normalization to vehicle treated cells and Combination index, using Webb’s fractional product method (Webb, 1963). An excel template was used to calculate the fraction affected from MTT data and the results were analyzed with the web version of Chalice Analyzer (Horizon Discovery) to calculate isobologram. pH was recorded before the addition of MTT through the measurement of phenol red absorbance spectra. The wavelength range was 350–650 nm with a step width of 5 nm and a bidirectional mode was employed for the reading. The path length correction, considering the volume (200 μL) and the thickness of the plate was taken into account, using appropriate options on the ClarioStar reader. An excel template was utilized to calculate the 560/440 nm ratio and the formula log$\left[\frac{560\text{ nm}}{\frac{440\text{ nm}}{0.0002}}\right]$/1.18 was employed to obtain pH values. Each treatment was performed in duplicate; experiments were repeated three times on cultures from different platings.

### Clonogenic Assay

A 1000 cells were seeded in a 6-well plate. After 72 h treatment with 3 μM of MDIVI-1 alone and in combination with 10 mM 2DG, fresh medium was added in each well and colonies were growth for 7 days. Cells were then fixed in 4% PFA for 10 min at room temperature and stained with crystal violet (0.5% in 1× PBS). Plates were scanned on a CanoScan LiDE 80 (Canon) at a resolution of 1200 dpi. Images were then cropped with ImageJ and analyzed with OpenCFU software (Geissmann, 2013). Experiments were repeated three times on cultures from different platings; each treatment was performed in triplicate during every experiment.

### Flow Cytometry

Cells were seeded on a 24 well plate at a density of 6^∗^10^4^ cells for well and treated with vehicle, 10 mM 2DG, 3 μM MDIVI-1, and combination treatments. After incubation time (72 h) cells were collected by tripsinization and stained with Annexin V-FITC and PI (Biovision) for 20 min at room temperature in dark condition and analyzed using a CyFlow ML (Partec) flow cytometer and FloMax software. A minimum of 10,000 events were recorded for each sample. Each treatment was performed in triplicate; experiments were repeated three independent times.

### Statistical Analysis

Data are given as means ± SD (standard deviation). Correlations were assessed using Spearman’s rank correlation analysis. For statistical comparison, two-way analysis of variance (ANOVA) or one-way analysis followed by Tukey’s *post hoc* test were employed. *P* < 0.05 were considered to be statistically significant.

## Results

### Mitochondrial Fission and Complex I Inhibitor MDIVI-1 Decrease Mitochondrial ATP Production and Bioenergetics

**FIGURE 1:**
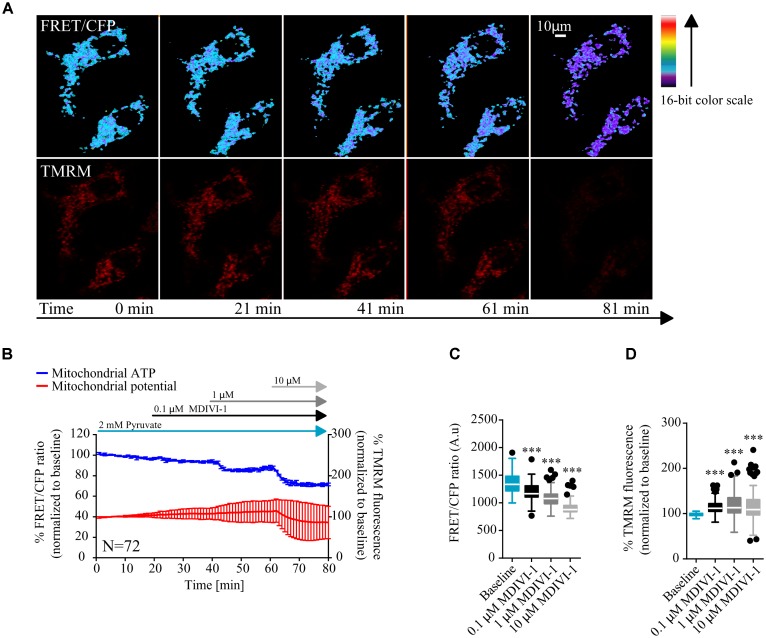
MDIVI-1 treatment decrease mitochondrial ATP production. **(A)** Representative images of the FRET/CFP ratio in MCF7 cells transfected with the mito ATeam FRET probe in MDIVI-1 titration experiments. TMRM was used to measure mitochondrial membrane potential changes. **(B)** Mitochondrial ATP and membrane potential kinetics in MCF7 during MDIVI-1 titration. FRET/CFP ratio and TMRM fluorescence kinetics were recorded simultaneously. Baseline was recorded for 20 min, after which 0.1, 1, and 10 μM of MDIVI-1 was added to the medium with intervals of 20 min. All data represent mean ± SD from *n* = 3 independent experiments and both signals are normalized to the baseline levels. **(C)** The absolute FRET/CFP ratio was analyzed by taking the minimal value reached by the probe in each cell after each MDIVI-1 addition into account. Values were evaluated by one-way ANOVA with Tukey post-test for multiple comparison (^∗^ indicates a *p*-value < 0.05, ^∗∗^ indicates a *p*-value < 0.01, and ^∗∗∗^ indicates a *p*-value < 0.001). **(D)** TMRM intensity values, normalized to the baseline levels, were analyzed by taking the maximal value reached during MDIVI treatments into account and statistical analysis was performed as described in **(C)**.

MCF7 cells were transfected with a mitochondrial ATeam expression vector, a FRET based sensor that enables the measurement of ATP production/consumption kinetics in living cells (Imamura et al., 2009). Cells were placed in Krebs buffer (KB) in the presence of 2 mM pyruvate to supply mitochondrial respiration. Following measurement of baseline kinetics for mitochondrial ATP and membrane potential, MDIVI-1 was titrated by adding increasing concentrations of this inhibitor, every 20 min (0.1, 1, and 10 μM). As highlighted in **Figures 1A,B** sequential additions of MDIVI-1 decreased mitochondrial ATP levels over the time-course, with 1 and 10 μM showing the highest effect. We analyzed the FRET/CFP ratio and found a significant decrease in the mitochondrial ATP production at all concentration used (**Figure 1B**). Interestingly, we also observed an increase in mitochondrial membrane potential when 0.1 and 1 μM of MDIVI-1 was used (**Figures 1A–C**). This is in line with a previous work that highlighted mitochondrial hyperpolarization following complex I inhibition with rotenone (Forkink et al., 2014). However, we recorded a high heterogeneity of changes in the membrane potential when 10 μM of the inhibitor was added, implying disruption of mitochondrial activity at different levels (**Figures 1A,B,D**).

### MDIVI-1 Treatment Decrease Intracellular Glucose Concentration

**FIGURE 2:**
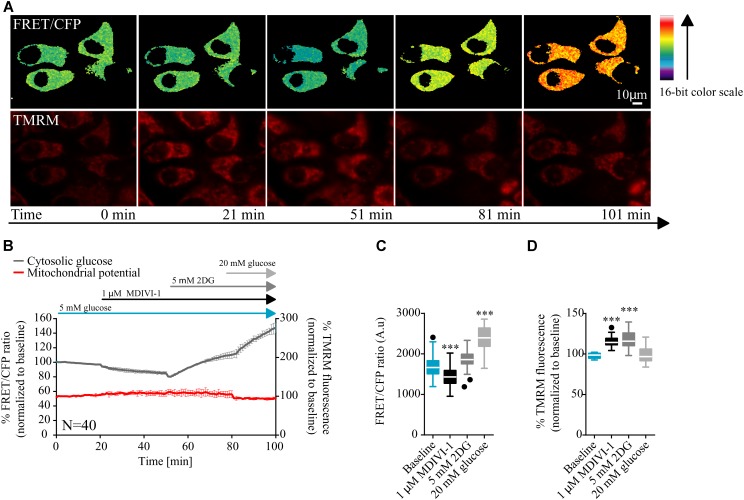
MDIVI-1 treatment decrease glucose consumption. **(A)** Representative images of the FRET/CFP ratio of MCF7 cells transfected with the glucose FRET probe. **(B)** Cytosolic glucose and membrane potential kinetics in MCF7 during MDIVI-1 treatment. FRET/CFP ratio kinetics and TMRM fluorescence were recorded simultaneously in MCF7 cells. Baseline was recorded for 20 min, after which 1 μM of MDIVI-1 was added to the medium. After 30 min 5 mM 2DG was added for a further 30 min, followed by the addition of 20 mM glucose for 20 min. All data represent mean ± SD from *n* = 3 independent experiments and both signals are normalized to the baseline levels. **(C)** The absolute FRET/CFP ratio was analyzed by taking the minimal value reached by the probe in each cell after each MDIVI-1 addition and the maximal value after 2DG and glucose treatment into account. Values were evaluated by one-way ANOVA with Tukey post-test for multiple comparison (^∗^ indicates a *p*-value < 0.05, ^∗∗^ indicates a *p*-value < 0.01 and ^∗∗∗^ indicates a *p*-value < 0.001). **(D)** TMRM intensity values, normalized to the baseline levels, were analyzed by taking the maximal value reached during MDIVI-1 treatments into account and statistical analysis was performed as described in **(C)**.

In order to have a better understanding of the bioenergetics status of the cell, we also studied the kinetics of glucose consumption, using a glucose sensitive FRET probe (Takanaga et al., 2008). In this case, cells where placed in 5 mM glucose in order to mimic a more physiological tumor environment. After measurement of baseline kinetics, we added 1 μM of MDIVI-1 and followed the intracellular glucose levels. As shown in **Figures 2A–C** addition of 1 μM MDIVI-1 significantly decreased cytosolic glucose kinetics. In order to confirm that the effects observed were dependent on glycolysis, we used the glycolytic inhibitor 2DG. We found that the addition of 5 mM 2DG increased cellular glucose concentration (**Figure 2B**). The further addition of 20 mM glucose caused an increment in the signal (**Figure 2C**). Again, we observed an increase in TMRM signal after 1 μM MDIVI-1 treatment that was maintained following 2DG addition. Intriguingly, addition of 20 mM glucose decreased membrane potential level to normal (**Figures 2A,B,D**). These findings suggested that upon induced mitochondrial dysfunction, in the presence of oxygen, the Warburg effect sustains cancer cells and also reveals a fast response and adaptability of breast cancer cells to changes in the surrounding environment.

### The Dual Inhibition of Glycolysis and OXPHOS as a Potential Treatment Strategy for Breast Cancer

Targeting the Warburg effect with 2DG has been proposed as a promising treatment strategy for a variety of cancer types (Aft et al., 2002; Zhang et al., 2006, 2014). 2DG is a glucose analog in which an atom of hydrogen replaced the 2-hydroxyl group. Upon cellular uptake, 2DG is phosphorylated by HKII with the formation of 2DG-P, which is not further converted in fructose-6-phosphate by phosphohexose isomerase. This ultimately leads to raised 2DG-P levels, hexokinase II inhibition and decreased cytosolic ATP (Maher et al., 2004). Due to observed decrease in mitochondrial ATP production and increase in glucose consumption following MDIVI-1, we further explored the idea of combining this treatment with glycolysis inhibition through 2DG.

**FIGURE 3:**
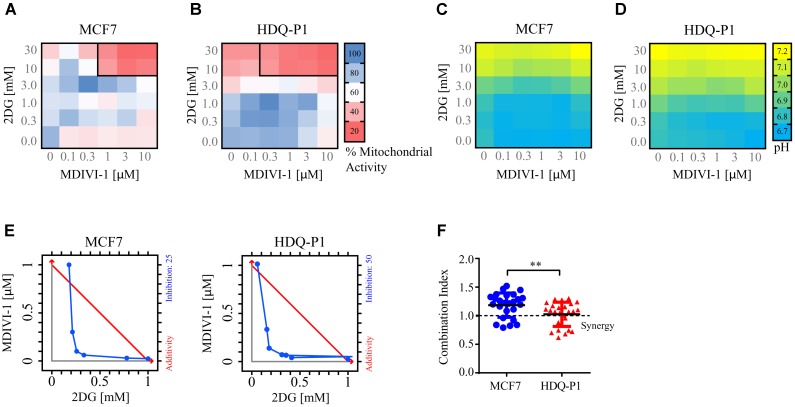
Combination treatment of 2DG with MDIVI-1 synergistically alters mitochondrial activity and pH levels in breast cancer cell lines. A 6 × 6 dose matrix assay was performed treating MCF7 and HDQ-P1 cells with increasing concentration of 2DG in combination with increasing concentration of MDIVI-1 for 72 h. Mitochondrial activity was measured by MTT assay, while the extracellular pH was measured by phenol red absorbance. Data are represented as a heatmap. **(A,B)** Mitochondrial activity values for 2DG in combination with MDIVI-1 in MCF7 and HDQ-P1 cells, respectively. **(C,D)** pH data for 2DG in combination with MDIVI-1 in MCF7 and HDQ-P1 cells, respectively. All values represent mean from *n* = 3 independent experiments. Each experimental treatment was performed in duplicate. **(E)** Isobologram analysis for fraction affected in % calculated from MTT, results for MCF7 and HDQ-P1 cells. **(F)** CI values were calculated using Webb’s fractional product method and analyzed with one-way ANOVA with Tukey post-test to test significance in MCF7 and HDQ-P1 cells, respectively (^∗∗^ indicates a *p*-value < 0.05). A CI value lower than 1 means synergy while a CI lower than 0.3 is classified as strong synergy; CI values > 1 are considered as antagonistic. Results represent means ± SD.

When breast cancer cells were treated with combination of 2DG and MDIVI-1, we recorded altered bioenergetics responses (**Figure 3**). We performed a 6 × 6 dose matrix format to test for any synergistic activity between MDIVI-1 and 2DG in either ER+ MCF7 or TNBC HDQ-P1 cancer cells. After 72 h treatment with increasing concentrations of 2DG in combination with increasing concentrations of MDIVI-1, mitochondrial activity was evaluated and data analyzed using Webb fractional product method (Webb, 1963) and isobologram analysis for evaluation of drug interactions. Additionally, we also measured the medium pH through phenol red absorbance as a read-out of lactate production during glycolysis. Higher concentrations of 2DG (10 and 30 mM) in combination with 0.3–10 μM MDIVI-1 induced a significant decrease in MTT absorbance in MCF7 cells (**Figure 3A**). Similar results were obtained for HDQ-P1 cells (**Figure 3B**), starting at lower concentrations (3–30 mM 2DG in combination with 0.3–10 μM MDIVI-1). We then analyzed the synergistic interactions between the treatments, and found that 10 and 30 mM 2DG highlighted synergistic combination index values (CI, **Figures 3A,B**, black boxes) in combination with 1–10 μM (MCF7 cells) and 0.3–10 μM MDIVI-1 (HDQ-P1). CI is an indicator of synergy (CI < 1), additivity (CI = 1) or antagonism (CI > 1). When using the isolobogram analysis, we were able to validate synergy between the two compounds in MCF7 and HDQ-P1 cells (**Figure 3E**). Moreover, the synergy interaction was more efficient in HDQ-P1 cells when compared to MCF7 (**Figure 3F**), as CI values were significantly lower.

As shown in **Figures 3C,D**, higher concentrations of 2DG (10 and 30 mM) increased the medium pH to 7 in both MCF7 and HDQ-P1 cells, respectively. Treatment with 10 and 30 mM 2DG in combination with MDIVI-1 (0.1–10 μM) also increased the pH at 7, indicating that cells started to take up lactate from the medium. Lower concentrations of 2DG (0.3–1 mM) in combination with MDIVI-1 (0.1–10 μM) did not change the pH compared to control conditions (**Figures 3C,D**).

**FIGURE 4:**
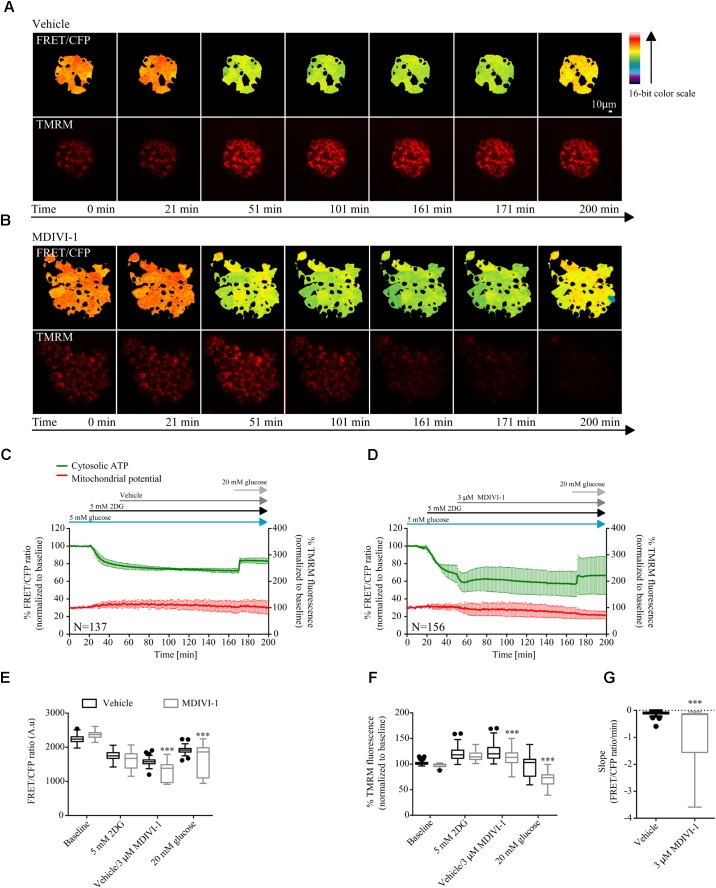
Combination treatment of 2DG with MDIVI-1 decrease ATP bioenergetics. **(A,B)** Representative images for FRET/CFP ratio from cytosolic ATP FRET probe and TMRM for vehicle and MDIVI-1 in combination with 2DG, respectively. **(C,D)** Cytosolic ATP and membrane potential traces in MCF7 during vehicle and MDIVI-1 treatment in combination with 2DG, respectively. FRET/CFP ratio kinetics and TMRM fluorescence were recorded simultaneously in MCF7 cells. Cells were placed in KB with 5 mM glucose and baseline was recorded for 20 min, after which 5 mM 2DG was added to the medium. After 30 min vehicle or 3 μM MDIVI-1 was added. After recording of the signal for 2 h 20 mM glucose was added for 30 min. All data represent mean ± SD from *n* = 3 independent experiments and both signals are normalized to the baseline levels. **(E)** The absolute FRET/CFP ratio was analyzed by taking the minimal value reached by the probe in each cell after 2DG and MDIVI-1 addition and the maximal value after 20 mM glucose treatment into account. Values were evaluated by one-way ANOVA with Tukey post-test for multiple comparison (^∗^ indicates a *p*-value < 0.05, ^∗∗^ indicates a *p*-value < 0.01 and ^∗∗∗^ indicates a *p*-value < 0.001). **(F)** TMRM intensity values, normalized to the baseline levels, were analyzed by taking the max and min values after each treatment into account and statistical analysis was performed as described in **(E)**. **(G)** Slope values were assessed by dividing the minimal FRET/CFP ratio to the Δtime (time offset – time onset). Values were analyzed using Mann-Whitney test to show significance (^∗∗∗^ indicates a *p*-value < 0.001).

Furthermore, we also took advantage of the cytosolic version of the ATP FRET reporter to study the bioenergetics response of MCF7 cell treated with the combination of 2DG and MDIVI-1. Cells were placed in 5 mM glucose and treated with 5 mM 2DG for 30 min after recording baseline values. As expected, addition of 2DG decreased the cytosolic ATP production (**Figures 4A–D**). When 3 μM MDIVI-1 was added to the medium, a further reduction of cytosolic ATP was observed when compared to vehicle treated cells (**Figure 4E**). The final addition of 20 mM glucose partially recovered ATP production; interestingly, this recovery was less pronounced in the combination treatment when compared to vehicle (**Figure 4E**). We also analyzed the slope of ATP consumption and found that MDIVI-1 treated cells showed faster ATP consumption kinetics when compared to vehicle treated cells (**Figure 4G**). Moreover 2DG increased the TMRM signal, while MDIVI-1 addition slightly decreased TMRM fluorescence when compared to vehicle (**Figure 4F**). Similar results were obtained when 20 mM glucose was added to cells under combination treatment (**Figure 4F**).

This suggested that combination treatments of 2DG and MDIVI-1 induced metabolic stress with an associated inhibition of glycolytic activity and mitochondrial respiration.

### 2DG in Combination With MDIVI-1 Decrease Clonogenic Potential and Increase Cell Death in Breast Cancer Cells

We next asked whether combination treatment would affect clonogenic potential and cell death levels of breast cancer cells. One of the optimal synergistic concentrations (10 mM 2DG in combination with 3 μM MDIVI-1) was subsequently selected to perform the experiments. Treatment of MCF7 cells with 10 mM 2DG induced a 30% decrease in colony formation, when compared to vehicle treated cells (**Figures 4A,B**). On the other hand, treatment with MDIVI-1 alone did not induce any change in colony formation (**Figures 4A,B**). Interestingly, 2DG/MDIVI-1 combination, showed a pronounced inhibition of colony formation, with a decrease to 7% of colonies when compared to vehicle or 2DG alone (**Figures 4A,B**). Similar results were observed in the TNBC cell lines where 2DG decreased the number of colonies to 70% and combination treatments to 5–10% when compared to either vehicle or 2DG treated cells (**Figures 4A,B**).

**FIGURE 5:**
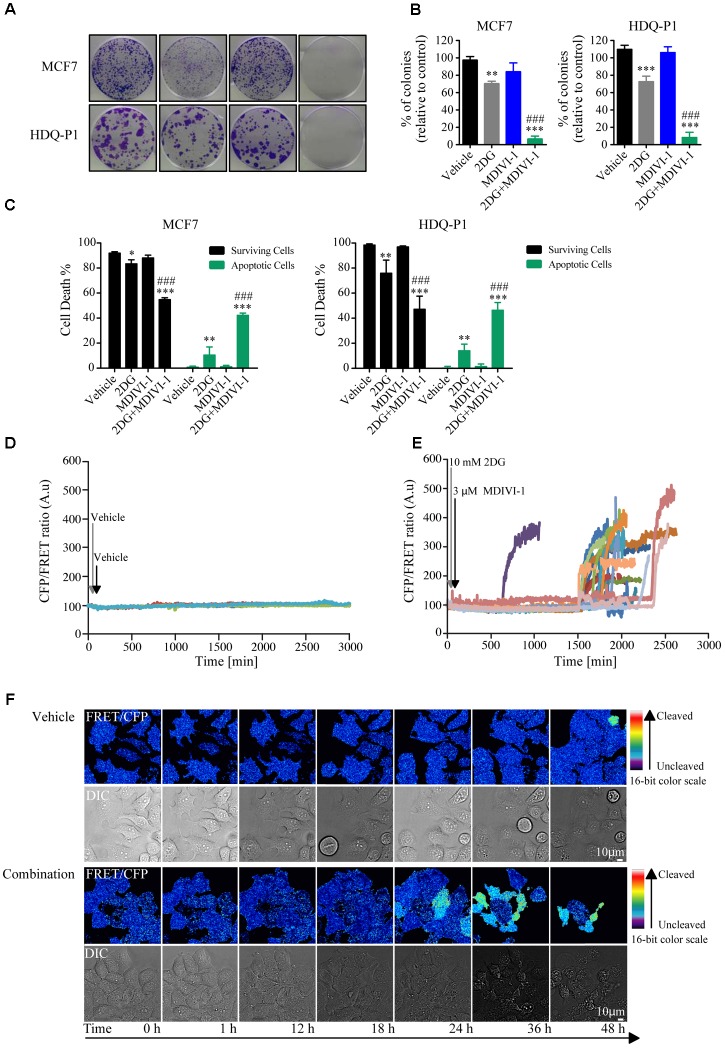
Combination treatment of 2DG with MDIVI-1 decrease clonogenic potential and surviving cell levels in MCF7 and HDQ-P1 cells. **(A,B)** Clonogenic assay of cells treated with vehicle, 10 mM 2DG, 3 μM of MDIVI-1 and combination treatment of 10 mM 2DG with 3 μM of MDIVI-1 in MCF7 and HDQ-P1 cells, respectively. After 72 h treatment medium was changed and clonogenic capability assayed after 7 days in culture. Images were cropped using ImageJ and colonies were counted automatically with Open CFU software and the change in colony growth was normalized to vehicle-treated cells. Bars represent means ± SD from three independent experiments. One-way ANOVA with Tukey post-test was used to assess significance comparison (^∗^ indicates a *p*-value < 0.05, ^∗∗^ indicates a *p*-value < 0.01, and ^∗∗∗^ indicates a *p*-value < 0.001). **(C)** Percentages of surviving (AnnV-/PI-) and apoptotic (Ann V+/PI- plus AnnV+/PI+ fraction) after vehicle, 10 mM 2DG or 3 μM MDIVI-1 alone and in combination in MCF7 and HDQ-P1 cells, respectively. Significance was assayed with a two-way ANOVA and Tukey post-test comparison (^∗^ indicates a *p*-value < 0.05, ^∗∗^ indicates a *p*-value < 0.01, and ^∗∗∗^ indicates a *p*-value < 0.001). Each column represents mean ± SD for *n* = 3 experiments. In all experiments, asterisk was used to indicate significance between treated conditions and vehicle control, while hash was used for significance between MDIVI-1 and 2DG. **(D,E)** Kinetics of caspase cleavage following treatment of MCF7-DEVD cells with vehicle or 10 mM 2DG in combination with 3 μM MDIVI-1. Cells were imaged for 48 h and treatment were added after the first 30 min (10 mM 2DG) and 60 min (3 μM MDIVI-1) of the experiment. An increase in the CFP/FRET ratio is indicative of caspase cleavage. **(F)** Representative images of caspase cleavage in MCF7-DEVD cells following vehicle or combination treatments.

Furthermore, we employed flow cytometry to assess the levels of Annexin V/PI levels after treatments. Following 72 h, addition of 10 mM 2DG slightly decreased surviving cells to 80% and increased apoptotic cell levels to 10–15%, while 3 μM MDIVI-1 treatment did not have any effect (**Figure 4C**). Interestingly, combination treatment decreased surviving cell levels to around 50–60% and increased apoptotic cells to 50–60% in both MCF7 and HDQ-P1 (**Figure 4C**). Finally, we took advantage of MCF7 cells overexpressing a caspase activity FRET reporter constituted by the cleavage sequence DEVD (Rehm et al., 2002). Cells were treated with vehicle or combination treatment of 10 mM 2DG and 3 μMDIVI-1. We found that during combination treatment, caspase activity increased following different kinetics (**Figures 5E,F**) when compared to control experiment (**Figures 5D,F**).

## Discussion

In this work, we demonstrated that the mitochondrial complex I and fission inhibitor MDIVI-1 inhibits mitochondrial ATP production, and increases glucose consumption in cancer cells. Furthermore, we highlighted that this inhibitor is synthetically lethal in breast cancer cells when combined with the glycolysis inhibitor 2DG.

We first determined the activity of MDIVI-1 on mitochondrial ATP production in breast cancer cells, by using a single-cell time lapse imaging approach. As previously stated, MDIVI-1 was found to inhibit OXPHOS complex I (Bordt et al., 2017). Thus, in line with these results, we found that titration of this inhibitor profoundly affected mitochondrial bioenergetics. Importantly, this effect occurred at much lower concentrations compared to the ones used to inhibit mitochondrial fission and complex I (25–100 μM) (Bordt et al., 2017). In conjunction with mitochondrial ATP experiments we also looked into the effect of this inhibitor on glucose consumption. Our data suggest that the decrease in mitochondrial ATP level and bioenergetics are accompanied by an activation of glycolysis as a compensatory mechanism to provide for the energetics need of breast cancer cells. Indeed, it has been previously shown that upon complex I inhibition with rotenone, breast cancer cells increase their glucose uptake and switch to a more glycolytic phenotype (Xu et al., 2015). Similar results were obtained with the gene-silencing of a mitochondrial complex I subunit (Suhane et al., 2013). Another independent study has shown that upon complex I inhibition with Metformin, glycolysis activates and promote cellular growth (Menendez et al., 2012).

Our study also demonstrates the importance of glycolysis inhibition as a strategy for the treatment of breast cancer. Glycolysis is an important metabolic route; in addition to function as a rapid source of ATP, it has been shown to be involved in other important metabolic pathways, such as pentose phosphate, hexosamine and glycogen synthesis (Hay, 2016). Cancer cells increase their glucose uptake by modulating the expression of hexokinase II (HKII), which, in turn, phosphorylates glucose and blocks its transport to the extracellular compartment mediated by specific transporters (Mathupala et al., 2001; Patra et al., 2013). HKII is associated with the mitochondrial outer membrane through the interaction with voltage-dependent anion-selective channel (VDAC). VDAC transfers the ATP produced by the mitochondria to HKII to catalyze the glucose phosphorylation reaction (Mathupala et al., 2006). A second regulation step is the conversion of phosphoenolpyruvate into pyruvate mediated by pyruvate kinases. In order to reroute metabolites to different pathways and support cell growth, low affinity pyruvate kinase M2 isoform is exploited by cancers to decrease this reaction (Israelsen and Vander Heiden, 2015). In conjunction, pyruvate is converted to lactate to maintain NAD^+^ levels. It has also been observed that acidification of tumor microenvironment by extracellular lactate may improve tumor invasion (Gatenby et al., 2006). Additionally, it has been highlighted that the microenvironment acidification and the competition for the available glucose by tumors, restricts the activity of the immune system (Chang et al., 2015; Ho et al., 2015). As previously stated, cancer cells have the ability to reroute metabolic pathways, hence this might contribute to attenuate the outcome of 2DG-based therapy. During glucose starvation or energetic stress, in order to survive, cancer cells switch to different sources of energy and carbon, through activation of AMPK signaling (Faubert et al., 2015). Furthermore, cancer cells might engage OXPHOS to compensate for decreased glycolysis or use alternative carbon sources. It has been shown that fatty acid oxidation increases upon glucose withdrawal to sustain ATP generation (Wolfe, 1998; Jelluma et al., 2006). Additionally, when glucose is removed and substituted by pyruvate, an increase in TCA cycle metabolites, alanine, and aspartate was observed (Oppermann et al., 2016). An alternative energy provider for cancer cells is glutamine, which is used to fuel TCA cycle or support nucleotide, protein, and lipid synthesis (De Vitto et al., 2016). Glucose starvation/deprivation might also activate glutaminolysis. Glutamine is initially deaminated by glutaminase with the production of ammonia and glutamate. Glutamate dehydrogenase converts this last one in α-ketoglutarate, to enter TCA cycle and produce ATP. In this context, it has been observed that glucose starvation increased activity of glutamate dehydrogenase (Jin et al., 2016). Hence the combined treatment of 2DG with an agent that inhibits mitochondrial respiration, as performed in this study, represents an attractive treatment approach.

2DG also activates autophagy, a conserved mechanism that recycles intracellular components, such as misfolded proteins or damaged organelles, and that has been proven to sustain cancer growth (Giammarioli et al., 2012; White, 2015). It has been proposed that autophagy activation upon 2DG treatment, involved ER stress and elicited a protective mechanism (Xi et al., 2011).

It has been also highlighted that 2DG-P accumulation in the cells increases the carbon flow into citrate production, impairing ADP phosphorylation, with a decrease in glycolysis (Pietzke et al., 2014). 2DG also acts on protein glycosylation and induces ER stress (Kang and Hwang, 2006; Kurtoglu et al., 2007). Hence other mechanisms beyond bioenergetics inhibition may contribute to the synergistic activity of 2DG and MDIVI-1 observed in this study. It has been demonstrated that 2DG sensitizes cancer cells to both chemotherapy and radiation therapy, showing that patients could benefit from this combination treatment (El Mjiyad et al., 2011). This glycolytic inhibitor has been tested in a variety of clinical trials, alone or in combination with chemotherapy (Raez et al., 2005; Goldberg et al., 2012; Sborov et al., 2015). It has been shown that 2DG is well tolerated up to a concentration of 200 mg/kg followed by whole brain irradiation (Mohanti et al., 1996). Hence combination treatments with 2DG represent a viable and promising strategy.

Indeed 2DG, as a single agent, has not yet been successfully translated to the clinic, due to poor efficiency recorded in clinical trials (Bost et al., 2016). As an example it has been shown that 2DG alone was not able to effectively remove cancer cells in an *in vivo* model of human osteosarcoma and non-small cell lung cancers (Maschek et al., 2004). In line with previous literature, we also found that only a small population of breast cancer cells treated with 2DG underwent cell death (**Figure 4C**). As pointed out in a review by Zhang et al. 2DG may have a cyto-protective effect, as ATP is crucial for the development of both intrinsic and extrinsic apoptosis (Zhang et al., 2014). When cells were treated with MDIVI-1 alone, cell death was also absent in ER+ (MCF7) and TN (HDQ-P1) breast cancer cells (**Figure 4C**), and only the combination was effective. Previous studies have also suggested that combining 2DG with agents that target OXPHOS represents a viable treatment strategy. Metformin inhibits complex I activity and increases glycolysis and lactate production (Chaube et al., 2015). Thus blocking lactate production was found to be deleterious for metabolism and synthetically lethal in melanoma (Chaube et al., 2015). Moreover, another group has reported a synthetically lethality of complex I inhibition with metformin and glucose withdrawal (Menendez et al., 2012). Of note, MDIVI-1 was effective at much lower concentrations and may have multiple mechanisms of action to inhibit mitochondrial respiration as a consequence of this dual targeting of complex I and fission inhibition and potentially other processes in mitochondria (**Figure 1**).

Metabolism and cellular bioenergetics are being recognized as important hallmark in different cancers pathways, such as formation of metastasis, tumor microenvironment, and treatment resistance (Pavlova and Thompson, 2016). To date, the current therapeutic landscape against this important module of cancer cells is lacking of options. Therefore, more targeted approaches that act on different metabolic/bioenergetics-related modules indeed need to be developed. This is especially important in light of the recent interest in the metabolic adaptation of immune cells in cancer progression (Xing et al., 2015). The integration of both fields has been shown to have a key role in cancer treatment (Renner et al., 2017).

Our experimental results also demonstrate, for the first time, that MDIVI-1 decrease mitochondrial bioenergetics at a concentration much lower of the one reported to inhibit fission. We also reported for the first time that the targeting of glycolysis and OXPHOS employing a combination of 2DG and MDIVI-1 can be applied to both ER+ and TNBC breast cancer subtypes. More importantly, it has to be pointed, that MDIVI-1 was found to possess low or no toxicity *in vivo* (Rappold et al., 2014). Unraveling new combination treatments and new drugs to target the “engines” of cancer cells have the potential to be critical in future investigations and to treat patients with therapy resistant cancer.

## Author Contributions

FL conceived and designed the study. FL and HD acquired the data. FL, HD, and JP wrote, reviewed, and/or revised the manuscript. JP supervised the study.

## Conflict of Interest Statement

The authors declare that the research was conducted in the absence of any commercial or financial relationships that could be construed as a potential conflict of interest.

## Abbreviations

2DG: 2-deoxy-D-glucose
ER: estrogen receptor
FRET: foerster resonance energy transfer
KB: krebs buffer
OXPHOS: mitochondrial oxidative phosphorylation
TMRM: tetramethylrhodamine methyl ester
TNBC: triple negative breast cancer.
